# Filamentous cyanobacteria triples oil production in seawater-based medium supplemented with industrial waste: monosodium glutamate residue

**DOI:** 10.1186/s13068-019-1391-1

**Published:** 2019-03-14

**Authors:** Liqun Jiang, Jiongming Sun, Changliang Nie, Yizhen Li, Jackson Jenkins, Haiyan Pei

**Affiliations:** 1 School of Environmental Science and Engineering, Shandong University, No.27 Shanda Nan Road, Jinan, 250100 China; 2 Shandong Provincial Engineering Centre for Environmental Science and Technology, No.17923 Jingshi Road, Jinan, 250061 China; 3 Department of Chemical and Biomolecular Engineering, Johns Hopkins University, Baltimore, MD 21218 USA

**Keywords:** Filamentous cyanobacterium, Biodiesel, *Spirulina subsalsa*, Seawater, Monosodium glutamate residue

## Abstract

**Background:**

To overcome the daunting technical and economic barriers of algal biofuels, we evaluated whether seawater can be a viable medium for economically producing filamentous *Spirulina subsalsa* as feedstock, using monosodium glutamate residue (MSGR) produced by the glutamate extraction process as an inexpensive nutrient source.

**Results:**

*Spirulina subsalsa* cannot grow in pure seawater, but exhibited faster biomass accumulation in seawater supplemented with MSGR than in freshwater medium (modified Zarrouk medium). Introducing seawater into media ensured this cyanobacterium obtained high lipid productivity (120 mg/L/day) and suffered limited bacterial infections during growth. Moreover, the yields of protein, carotenoids and phytols were also improved in seawater mixed with MSGR. *S. subsalsa* exhibited high biomass and lipid productivity in bag bioreactors with 5- and 10-L medium, demonstrating the potential of this cultivation method for scaling up. Moreover, seawater can produce more biomass through medium reuse. Reused seawater medium yielded 72% of lipid content compared to pristine medium. The reason that *S. subsalsa* grew well in seawater with MSGR is its proficient adaptation to salinity, which included elongation and desaturation of fatty acids, accumulation of lysine and methionine, and secretion of sodium. The nutrients provided by MSGR, like organic materials, played an important role in these responses.

**Conclusion:**

*Spirulina subsalsa* has an efficient system to adapt to saline ambiance in seawater. When supplemented with MSGR, seawater is a great potential medium to produce *S. subsalsa* in large scale as biofuel feedstock. Meanwhile, value-added products can be derived from the ample protein and pigments that can broaden the range of biomass application and improve this biorefinery economics.

**Electronic supplementary material:**

The online version of this article (10.1186/s13068-019-1391-1) contains supplementary material, which is available to authorized users.

## Background

Many studies have characterized the potential of microalgae as a feedstock for renewable biofuel and bioproducts, noting benefits such as high growth rate, high oil content, and little demand of arable land [1, 2]. Among these researches, unicellular microalgal species received most attention, such as *Scenedesmus*, *Chlorella*, and *Nannochloropsis* etc., due to their adaptation in wastewater and high lipid content [3]. However, in the process of biodiesel production, harvesting of unicellular microalgae requires a substantial amount of capital and energy because unicellular cells have low densities and microscopic dimensions (0.5–30 µm) that make their separation from culture difficult, especially in large scale [4, 5]. Filamentous cyanobacteria can usually form aggregates and be collected easily through filtration or flotation, which has evident advantages over unicellular algae in reducing cost of harvest. However, a minority of biofuel researches focused on filamentous cyanobacteria due to their low-level lipids (mostly less than 10%). While nitrogen depletion or high salinity was commonly employed as stresses to improving carbon flux towards lipid synthesis, a method to enhance the lipid accumulation of cyanobacteria is urgently needed [6].

To commercially produce cyanobacteria-based biofuel, it is necessary to reduce the demand of the cultivation on freshwater supply [7]. To address both of these requirements, inexhaustible seawater supplemented with wastewater highlights a promising way to save freshwater from medium preparation for cyanobacterial cultivation and trigger lipid production in biomass, as we pinpointed previously [8].

Limnetic filamentous cyanobacterial species are better suited to obtain increased lipid productivity from this new method than marine species that may not experience salt stress in seawater. Based on this, *Spirulina subsalsa* reserved by Institute of Hydrobiology (Hubei, China) is a good candidate. *Spirulina subsalsa* grow to a size of approximately 200 µm, facilitating harvest compared to smaller microalgae. Moreover, it was isolated from an alkaline hot spring and can tolerate 0–5 wt/vol% salinity [9], which indicated an osmoregulation mechanism in *S. subsalsa* aiding its adaptation to seawater. As of yet, there are few publications focusing on biofuel applications for *S. subsalsa*, due to the lower lipid content in this cyanobacterial cell. This would be an exciting development to demonstrate the feasibility of applying *S. subsalsa* biomass to produce biofuel using seawater and wastewater as a promising cultivation method.

Wastewater containing luxury macronutrients and trace heavy metals can benefit cyanobacterial growth and improve the production system. Hence, residue or byproducts derived from industrial food production can obtain a high score in terms of sanitation and nutrients.

Monosodium glutamate (MSG) as a flavor enhancer is extensively used in food products. China produces 75% of the world’s supply of MSG since 2011, according to data from the Chinese Condiments Association. During MSG production, there is residue generated after glutamate extraction and sterilization that is rich in nitrogen and phosphorus, with total nitrogen (TN) of 62–72 g/L and total phosphorus (TP) of 657–686 mg/L. Moreover, it contained trace heavy metals (0.0021–0.0019 mg/L cadmium, 0.0157–0.0199 mg/L cobalt, 0.0492–0.0521 mg/L chromium, 0.0451–0.0483 mg/L copper, 0.1100–0.1129 mg/L manganese). The residue (MSGR) may be an economical and acceptable nutrient source for *Spirulina* biomass production from seawater.

Based on this information, we chose seawater as our source of water and minerals for algal biomass production, and MSGR as the source of macroelements (nitrogen, phosphorus) to culture limnetic *S. subsalsa*. The main goals in this study comprise: (1) observing the growth and lipid accumulation of *S. subsalsa* in seawater media; (2) validating the potential of scaling up this new cultivation system; (3) improving the efficiency of seawater to produce cyanobacterial biomass as a biofuel feedstock; and (4) analyzing the regulation of limnetic *S. subsalsa* in response to seawater.

## Methods

### Microorganism species and cultivation medium source

*Spirulina subsalsa* was purchased from the Freshwater Algae Culture Collection of the Institute of Hydrobiology (FACHB-Collection), and cultured as FACHB recommended.

In the experiment, modified Zarrouk medium (mZM) was used as the control freshwater medium to culture *S. subsalsa*. This medium, as shown in previous study [10], included (in g/L): 2.5 NaNO_3_; 0.5 NaCl; 0.15 MgSO_4_·7H_2_O; 0.04 CaCl_2_·2H_2_O; 1.25 single super phosphate; 0.898 KCl; 16.8 NaHCO_3_.

The seawater was collected from Qingdao, China, and filtered through 0.22 μm membranes before use. It contained: 76.38 ± 7.16 mg/L TOC, 0.60 ± 0.00 mg/L NH_3_-N, 3.67 ± 0.14 mg/L TN, 0.003 ± 0.000 mg/L TP, 13.92 ± 0.14 g/L Na, 346.6 ± 7.2 mg/L K, 988.8 ± 13.6 mg/L Ca, 1295 ± 3 mg/L Mg, 24.10 ± 3.58 mg/L Al, 5.60 ± 0.08 mg/L Fe, 5.20 ± 0.06 mg/L Cu, 2.61 ± 0.08 mg/L Zn; Mn and Co were not detected.

The nutrient MSGR is a residue remaining after glutamate extraction and bacteria removal during MSG production, and was supplied by Liangshan Linghua Gourmet Powder factory (Jining, China). The brown acidic liquid MSGR was used directly for algal cultivation and presented the following characteristics: COD_Cr_ 18–22 g/L, BOD_5_ 10–11 g/L, TOC 6.9–7.0 g/L SO_4_^2−^ 190–210 mg/L, NH_3_-N 550–575 mg/L, TN 62–71 g/L, and TP 657–686 mg/L, pH 3.8-4.3.

### Experimental runs

This study was designed as below to check the feasibility of seawater to produce biomass as biofuel feedstock in low cost, which is schematically shown in Additional file 1: Fig. S1.

#### Optimizing MSGR loading

Eight ratios of MSGR to seawater (1/1000, 1/500, 1/200, 1/100, 1/50, 1/25, 1/10 and 1/5, *V*_MSGR_/*V*_seawater_) were made up as the seawater media. Pure seawater, mZM and MSGR were employed as controls (Additional file 1: Fig. S1a).

*Spirulina subsalsa* reaching the late exponential phase was recovered by centrifugation and washed three times with distilled water, then suspended in distilled water. To get a homogeneous inoculum free of nutrients, the microalgal suspension was vortexed before inoculation.

Triangular flasks (250 mL) sealed with *Parafilm* were used as the batch containers. The prepared inoculum was inoculated into 150 mL medium in the container with an initial biomass concentration of 0.1 g/L, and cultivated under conditions similar to those of our previous study: 25 ± 1 °C and light intensity of 60 µmol/m^2^/s for 24 h every day [10]. All the batch experiments were conducted in triplicate.

#### Scaling up with bag reactor and reused medium

Culturing *S. subsalsa* in bag reactor was a preliminary step to scale up this cultivation system (Additional file 1: Fig. S1b). Baggy bioreactor (Additional file 1: Fig. S1c) is a kind of closed reactor that can limit evaporation and contamination, and is economical and widely useable, compared to other reactors made of polymethyl methacrylate. Two medium volumes (5 L and 10 L) were used to check the growth of *S. subsalsa* in baggy reactor (0.58 m × 0.45 m).

To obtain as much biomass from per unit of seawater as possible, the growth of *S. subsalsa* in reused medium was also observed, as the schematic figure shown (Additional file 1: Fig. S1b).

### Determination of growth of *Spirulina subsalsa*

The growth of *S. subsalsa* was measured every 24 h and expressed as dry cell mass (DCM) and chlorophyll a content. Measurement of dry cell mass was carried out as Jiang et al. [10] did, according to the procedure: 5 mL culture was centrifuged, washed three times with DI water, dried under 60 °C and weighted for obtaining biomass concentration.

For cyanobacteria in baggy reactor, areal productivity (*P*_A_) and volumetric productivity (*P*_V_) were calculated. The former was based on the place occupied by the reactor, while the latter was considered the input of medium:

1 $$P_{\text{A}} = {\text{ DCM }}/ \, \left( {A \times T} \right)$$

2 $$P_{\text{V}} = {\text{ DCM }}/ \, \left( {V \times T} \right)$$

where *A* is the area of baggy reactor, *V* is the volume of medium, and *T* stands for the period of cultivation.

Chlorophyll a (chl a) was detected using the same method from our previous report [11]. Briefly, 1 mL culture was harvested by centrifugation. After discarding the supernatant, 1.5 mL of methanol (99%) was added to the pellet, mixed well with vortex and immersed in the dark for 24 h at 45 °C. The chlorophyll extract was obtained through centrifugation at 4000 rpm for 10 min. After measuring the optical density (absorbance at wavelength of 665.2 and 652.4, *A*_665.2_ and *A*_652.4_) of the extract, chl a concentration can be calculated as:

3 $${\text{Chl}}\;{\text{a}}\left( {{\text{mg}}/{\text{L}}} \right) \, = \, 16.72 \, A_{665.2} {-} \, 9.16A_{652.4}$$

### Analysis of metabolites in biomass

Based on the growth curve, *S. subsalsa* was harvested by filtration with 200-mesh bolting silk at the point of maximum biomass concentration. Collected cyanobacterial biomass was dried to constant mass at − 50 °C in a lyophilizer (EYELA FDU-1200, Tokyo Rikakikai, Japan), and then ground to homogeneous powder in preparation for nutritional materials analysis.

#### Determination of total lipid content and fatty acid profiles

Total crude lipid of cyanobacterial biomass was quantified gravimetrically following the extraction. About 0.1 g dried biomass powder was transferred into a 5 mL chloroform/methanol (2:1, v/v) mixture and homogenized for 10 min under a sonication; this was followed by centrifugation at 4000 rpm at 4 °C for 10 min to collect supernatant in a separation funnel; the entire extraction process was repeated twice; sodium chloride solution (0.9%) was then added at a proportion of 1:5 (v/v) of lipid extract; the extract was shaken vigorously for 1 min and allowed to undergo phase separation for 15 min; the lower phase containing essentially all the extracted lipids was transferred into a clean weighed glass tube and dried under nitrogen flow at 60 °C to get the weight of lipids for calculating total lipid content in cells.

The fatty acids and phytols in *S. subsalsa* were in situ esterified with methanol for GC–MS detection, which was accomplished through a pre-esterification process-catalyzed fatty acids in cells by Amberlyst-15 and the following transesterification base catalyzed by 4 wt% KOH/methanol in a 60 °C water bath. Then, fatty acid methanol ester (FAME) was collected in 1 mL hexane in the upper layer and separated from water and other residues staying in the lower layer. The upper layer solution (100 μL) was transferred into a GC vial with fused-in insert, and 25 μL heptadecanoic acid methyl ester (C17: 0, 2 mg/mL) was added as internal standard of methyl ester. The FAME profile was analyzed by gas chromatography–mass spectrometry (GC–MS, Trace GC ultra and DSQ II) equipped with an automatic sampler (Thermo Fisher, USA) and a capillary column of VF–23 ms (30 m × 0.25 mm × 0.25 μm, Agilent). The column started at 150 °C and held for 1 min, raised to 165 °C at a rate of 1 °C/min. The FAME composition was expressed as its percentage compared with the total transesterifiable lipids of biomass.

To evaluate the biodiesel property, the average degree of unsaturation (ADU), kinematic viscosity, specific gravity, cloud point, cetane number (CN), iodine value and higher heating value (HHV) were calculated to follow the Eqs. (4–10) in the report of Song et al. [12]:

4 $${\text{ADU}} = \sum M \times Y_{i}$$

5 $$y_{1} = - 0.6316x + \, 5.2065 \, R^{2} = 0.6704$$

6 $$y_{2} = 0.0055x + \, 0.8726 \, R^{2} = 0.6644$$

7 $$y_{3} = - 13.356x + \, 19.994 \, R^{2} = 0.6809$$

8 $$y_{4} = - 6.6684x + \, 62.876 \, R^{2} = 0.8049$$

9 $$y_{5} = 74.373x + \, 12.71 \, R^{2} = 0.9484$$

10 $$y_{6} = 1.7601x + \, 38.534 \, R^{2} = 0.38$$

where *M* is the number of carbon–carbon double bonds in each FA composition, *Y*_i_ is the mass fraction of the corresponding FA constitution, *y*_1–6_ represent kinematic viscosity, specific gravity, cloud point, cetane number, iodine value and higher heating value successively, and *x* is the value of ADU.

#### Determination of carbohydrate

The carbohydrate in biomass was quantified by colorimetric assay on a 96-well plate in a Multiskan FC (Multiskan FC, Thermo, USA) at 620 nm wavelength, following extraction from about 100 mg biomass using hydrochloric acid, as we previously did [10]. Around 100 mg biomass was reacted with 15% hydrochloric acid in a 50 mL triangular flask under a vigorously boiling water bath for 10 min. Allowing the liquid cooling down, centrifugation (4000 rpm, 10 min) was employed to get supernatant free of particles than the extracted soluble carbohydrate. An aliquot of 1 mL supernatant was transferred into a clean glass vial with screw cap and incubated for 10 min in a vigorously boiling water bath after adding 4 mL anthrone (2 wt% H_2_SO_4_). Then, the vial was placed into ice bath for instantly cooling down. The last step was colorimetrically determining the absorbance in a Multiskan FC. Solutions containing different level of glucose were used to set a relationship between sugar concentration and absorbance.

#### Determination of protein content and amino acid profiles

Characterization of protein content in algal biomass involved elemental analysis of total N [13], and the use of an ‘appropriate’ nitrogen-to-protein conversion factor (4.78) [14]. Specifically, about 50 mg algal powder was digested with CuSO_4_·5H_2_O, K_2_SO_4_ and H_2_SO_4_, and then N concentration was measured for the calculation of protein content.

The amino acid composition of *S. subsalsa* was obtained with an amino acid analyzer (L-8900, Hitachi, Japan).

#### Determination of carotenoids

As described by Shao et al. [15], the carotenoid content was measured with the solution extracted from the algal culture, which was conditioned by ethanol (95%, v/v). Tested biomass was collected by centrifugation, suspended with 3 mL ethanol (95%, v/v), disrupted ultrasonically, and then incubated in the dark for 12 h. The extracted solution was obtained through centrifuging samples from the last procedure, and absorbance at 450 nm was measured with a UV–vis. spectrophotometer (UV-2450, Shimadzu, Japan). Eventually, the carotenoid contents (CC, g/L) were estimated with this equation:

11 $${\text{CC }} = \, A_{450} \times \, 3 \, / \, 250$$

where *A*_450_ is the absorbance at wavelength of 450 nm, 3 is the ratio between the volume of ethanol addition and culture used or carotenoids detection, and 250 represents the conversion factor calculating absorbance to CC.

### Determination of intracellular inorganic elements

The measurement of mineral and trace elements was conducted with an atomic absorption spectrophotometer (180-80, Hitachi, Japan). The pretreatment of algal biomass was similar to that used by Tibbetts et al. [16]. About 100 mg of sample was digested by concentrated HNO_3_ at 95 °C for 75 min, after which organic matter was dissolved with the addition of H_2_O_2_ (30% v/v). Samples were then incubated at 95 °C for 1 h in concentrated HCl, and made up to the required volume with ultrapure water, awaiting detection.

### Statistical analysis

Results are presented in the form of mean values ± standard deviation from three independent experiments and analyzed using one-way analysis of variance in Duncan’s test. A difference was considered statistically significant when *p* < 0.05.

## Results and discussion

### The viability and lipid production of *Spirulina subsalsa* in seawater with nutrients supplemented by MSGR

#### Chlorophyll and biomass accumulation

**Fig. 1:**
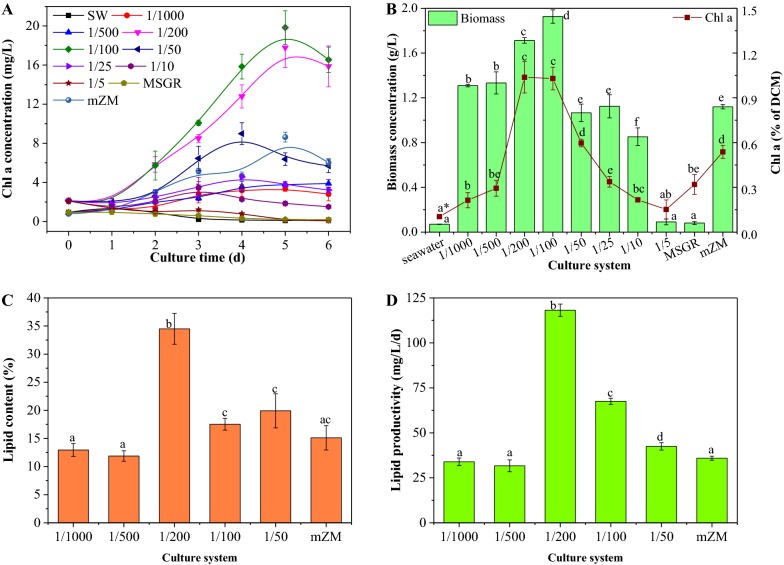
The growth and lipid production of *Spirulina subsalsa* grown in mZM, in seawater, in MSGR, and in seawater supplemented with MSGR in different volume ratio (*V*_MSGR_/*V*_seawater_) from 1/1000 to 1/5, **A** daily chlorophyll accumulation, **B** maximum biomass concentration and corresponding chlorophyll content in dry biomass, **C** lipid content, **D** lipid productivity. All data are averages of biological triplicates ± standard deviation. *Data for the same parameter followed by different letters are significantly different by Duncan’s test at *p* < 0.05

The growth of *S. subsalsa* was compared between those grown in cultures supplied with a range of MSGR concentrations and those grown in three control cultures, which composed solely of seawater, MSGR, or modified Zarrouk medium (mZM), respectively (Fig. 1). In the seawater and MSGR controls, no increment in chlorophyll a (chl a) synthesis was observed and algae died gradually by the 3rd or 4th day (Fig. 1A). *S. subsalsa* could not survive in the 1/5 treatment either, and manifested decreases in chlorophyll concentration from the beginning. Indeed, except for the 1/50, 1/100 and 1/200 loadings, the chl a levels observed in the seawater-based media were all lower than that in mZM. A considerable improvement of chlorophyll a levels was consistently seen for the 1/100 and 1/200 groups throughout the duration of the culture.

As chlorophyll is the main element related to the photosynthetic system in cyanobacteria, Fig. 1B presents the proportion of chl a in dry cell mass, for a more direct photosynthesis capacity comparison (regardless of varying biomass levels) between algae growing in different media. *Spirulina* usually contains 0.8–2% chl a in dry biomass [17]. In the mZM control, *S. subsalsa* possessed 0.77% chl a. Other than the 1/100 and 1/200 MSGR media, we found that the seawater media and the MSGR control significantly depressed chl a accumulation in *S. subsalsa* (*p *< 0.05). Conversely, cells in 1/100 and 1/200 MSGR media obtained a higher chl a level than in the mZM control.

The chlorophyll concentration suggested the capability of growing photoautotrophic microalgae for biomass production (Fig. 1). After 5 days of growth, excellent biomass concentrations (1.9 and 1.7 g/L, respectively) were achieved for the 1/100 and 1/200 MSGR seawater media, higher than with other loadings (less than 1.3 g/L) and higher still than in the mZM control (1.1 g/L).

Extracellular particles in culture could also have an impact on the photosynthesis system. Cultivation of *S. subsalsa* in mineral-enriched seawater and sulfate-enriched MSGR always resulted in the formation of white precipitates, impending the passage of light to the algal cells. This phenomenon occurred severely for high ratios of MSGR in seawater, such as 1/25, 1/10 and 1/5, whereas it was slight in treatments with the other, lower MSGR concentrations. These results are consistent with the growth profiles of *S. subsalsa* in the respective MSGR concentrations, indicating that the precipitate formation inhibited the biomass production through decreasing photo-availability for *S. subsalsa*.

#### Lipid production

Lipid yield was analyzed from 1/1000 to 1/50 MSGR and mZM control (Fig. 1B, C), because little biomass was collected from higher MSGR concentrations and other controls. Biomass from 1/25 and 1/10 runs was not considered due to too much precipitate formulation during *S. subsalsa* growth. *S. subsalsa* usually has a low lipid production (around 15%) in mZM, similar to our previous result [10]. Cells in seawater media (1/1000, 1/500, 1/100 and 1/50 MSGR) obtained the same level of lipid content to that in mZM; however, the 1/200 MSGR produced biomass with the highest lipid content with 35% (Fig. 1C). This lipid content can compete with most green algae that have lipid content around 25–30% [3].

The energy for biosynthesizing lipids originates from the light captured and transferred by chlorophyll, which indicates possibly higher lipid production of biomass in 1/200 and 1/100 MSGR due to their much higher chlorophyll a content shown in Fig. 1A, B. However, the destination of photosynthetic energy, along with carbon fixed, is usually driven to lipid or carbohydrate by environmental stress, like nutrient deficiency, extreme temperature, salinity, etc. [6]. In this case, *S. subsalsa* shared the same culture condition of temperature, light cycle and intensity and suffered from similar salt stress, but went through different nutrient availability. The salt stress was mostly led by seawater containing 17 g/L of cation that was around three times higher than 6 g/L in mZM. The main elements of nitrogen and phosphorus that supplied by MSGR in 1/200 MSGR (NH_3_-N 2.7 mg/L, TN 330 mg/L, TP 3.2 mg/L) were only approximately half of that in 1/100 MSGR (NH_3_-N 5.7 mg/L, TN 670 mg/L, TP 6.8 mg/L). After culturing *S. subsalsa*, the ammonia and TP were exhausted, and the remaining TN was 143 mg/L and 142 mg/L in 1/100 and 1/200 MSGR, respectively. These results suggest that the remaining nitrogen might not be bioavailable to *S. subsalsa,* and nitrogen and phosphorus limitation likely occurred in 1/200 MSGR. This difference in nutrient availability altered the energy storage of *S. subsalsa*. For *S. subsalsa* grown in 1/200 MSGR, energy was mainly stored as lipid (35% of lipid and 8% carbohydrate), but cells grown in 1/100 MSGR stored energy as carbohydrates (18% of lipid and 16% carbohydrate) (Fig. 1C and Additional file 1: Fig. S2).

The lipid yield from 1/200 MSGR suggests that this is approximately the appropriate wastewater dose in seawater with just enough nutrients for cells to reach a high biomass and just limited enough nutrients for triggering lipid synthesis with salt stress from seawater together, as we proposed [8]. Indeed, characterizing the energy absorption, carbon fixation and metabolic pathways of this cyanobacterium in seawater with different concentrations of MSGR represents a robust opportunity for future study of further improving lipid generation for commercial applications.

The lipid productivities from 1/1000 and 1/500 MSGR had no significant difference compared to the mZM control, while higher biomass concentration in 1/100 MSGR (Fig. 1B) led to higher lipid productivity than mZM control. Seawater with 1/200 MSGR addition was the most appropriate medium to achieve high lipid productivity (120 mg/L/day) that is 3.3 times higher than 36 mg/L/day in mZM.

Additionally, lipid quality was also evaluated as the part of consideration for biodiesel feedstock. Table 1 shows the data on kinematic viscosity, specific gravity and other four biodiesel properties achieved in 1/200 MSGR and mZM control. These values are calculated from fatty acids. The cyanobacterial biomass from seawater medium was suitable as biodiesel feedstock, because all six important biodiesel properties met the ASTM and EN biodiesel standards.

**Table 1:** The lipid production and fatty acid profile of *Spirulina subsalsa* in seawater supplemented with 1/200 MSGR (V_MSGR_/V_seawater_) (SW-MSGR), and in mZM

	SW-MSGR	mZM	ASTMD 6751-08	EN 14214
Biodiesel properties				
Kinematic viscosity 40 °C (mm^2^/s)	4.81 ± 0.02	4.85 ± 0.01	1.9–6.0	3.5–5.0
Specific gravity (kg/L)	0.88 ± 0.01	0.88 ± 0.01	0.85–0.90	0.86–0.90
CP (°C)	11.65 ± 0.19	12.50 ± 0.13		
CN	58.71 ± 0.08	59.14 ± 0.22	≥ 47	≥ 51
IV (gI_2_/100 g)	59.20 ± 2.04	54.42 ± 2.73		51–120
HHV (MJ/kg)	39.63 ± 0.01	39.52 ± 0.01		

All data are averages of biological triplicates ± standard deviation

### The advantage of seawater to culture *Spirulina subsalsa*

In addition to higher biomass and lipid accumulation of *S. subsalsa* in media prepared with seawater and MSGR, we also investigated other benefits that can be given by seawater. The behavior of *S. subsalsa* in different media was compared, including cell morphology and bioinvasion.

The morphology of *S. subsalsa* was observed under a microscope, as shown in Additional file 1: Fig. S3. Little cyanobacteria grew in seawater and MSGR control, so microscopy images were only taken for cells in 1/1000–1/50 MSGR media and mZM. In low loadings of MSGR (1/1000, 1/500), most filaments were short or around 100 µm and little bacteria grew (Additional file 1: Fig. S3a, b). Seawater media with 1/200 and 1/100 MSGR and mZM control cultured filaments longer than 200 µm (beyond camera sight) (Additional file 1: Fig. S3c, d, h). With increasing MSGR concentration in seawater to 1/50, 1/25 and 1/10, the length of cyanobacterial filament decreased sharply and bacteria density increased (Additional file 1: Fig. S3e–g). The high addition of MSGR led to bacteria propagating and broke the extension of cyanobacterial filaments (Additional file 1: Fig. S4). Therefore, the growth of *S. subsalsa* may depend on the elongation of filament. Meanwhile, appropriate addition of MSGR made *S. subsalsa* obtain filaments as long as that in mZM and ensured the feasibility and efficiency of biomass collection through filtration.

The above biomass, lipid and morphology results suggest that seawater is an excellent choice when producing *S. subsalsa* as biofuel feedstock, and prove that seawater cultivation could create benefits including growth and lipid production, and resistance to bacterial infections, as we previously expected [8]. Moreover, cells in long filaments, like *S. subsalsa*, were readily collected through filtration. The combination of seawater and *S. subsalsa* exemplifies the production of the algae-based biodiesel.

### Diverse potential application of *Spirulina subsalsa* biomass

In addition to biodiesel yield, other alternative uses for biomass can output co-products that can enhance the economic feasibility of algal-based commodities [2]. To realize this goal, the contents of protein, phytols and inorganic elements (minerals and trace elements) were detected as potential factors for animal feeds or agricultural fertilizers.

#### Protein accumulation

**Fig. 2:**
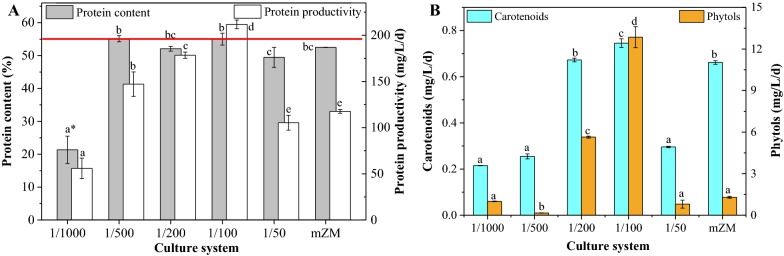
Protein (**A**) and carotenoids and phytols (**B**) accumulation for *Spirulina subsalsa* grown in mZM and in seawater supplemented with MSGR in different volume ratios (1/1000, 1/500, 1/200, 1/100 and 1/50, *V*_MSGR_/*V*_seawater_). The red line stands for a protein content of 55% from food standard GB 16919 [28]. All data are averages of biological triplicates ± standard deviation. *Data for a given parameter followed by different letters are significantly different by Duncan’s test at *p* < 0.05

Protein is the main macromolecular component in most cyanobacterial cells and has very high nutritional value for animal feed or pharmacy. Total concentrations of protein, expressed as percentages of DCM, in the biomass harvested after 5 days of growth are presented in Fig. 2A. Cyanobacteria in the 1/500, 1/200, 1/100 and 1/50 groups produced similar level of protein to that in mZM (*p *> 0.05). In the 1/500 and 1/100 groups, the protein content matched with the standard of more than 55% [18]. The maximum protein content occurred in the treatment with 1/100 MSGR in seawater.

#### Carotenoids and phytols production

Carotenoids and phytols are important components of chlorophyll, and play crucial roles in photosynthesis, antibacterial activity [19], and chemical defense against herbivory.

Carotenoids are used in the food industry and are unique constituents of a healthy diet, playing an important role in improving antioxidant status and preventing cancers [20]. As shown in Fig. 2B, the ratios of 1/100 and 1/200 between MSGR and seawater possessed superiority as media for carotenoids production due to their higher biomass productivities than mZM.

These two ratios of MSGR in seawater also improved the phytol production from *S. subsalsa*. Biomass in 1/100 MSGR and seawater had the highest phytol productivity of 12.9 mg/L/day, followed by 5.6 mg/L/day in the 1/200 group, which all outweighed the 1.3 mg/L/day level in mZM.

#### Minerals and trace elements

Minerals and trace elements are of pivotal importance for animals and plants because they cannot synthesize the materials and must depend on uptake from feeds or fertilizers. We evaluated the inorganic materials in *S. subsalsa* biomass to explore its application in livestock and agriculture from both the nutritional and the toxicological point of view.

Minerals assayed include calcium (Ca), magnesium (Mg) and potassium (K). Trace elements assayed include cobalt (Co), copper (Cu), iron (Fe), manganese (Mn), selenium (Se), vanadium (V) and zinc (Zn). Heavy metals assayed include arsenic (As), cadmium (Cd), chromium (Cr) and lead (Pb). The results obtained for the minerals and trace elements analyzed in all the cyanobacterial samples from different media are presented in Table 2.

**Table 2:** Elemental composition (DCM basis) of *Spirulina subsalsa* cultivated in mZM and seawater supplemented with MSGR in different volume ratios (1/1000, 1/500, 1/200, 1/100 and 1/50, *V*_MSGR_/*V*_seawater_)

	1/1000	1/500	1/200	1/100	1/50	mZM
Minerals (%)						
Calcium	1.14 ± 0.01	3.06 ± 0.04	4.58 ± 0.03	2.92 ± 0.01	3.53 ± 0.02	1.00 ± 0.02
Magnesium	2.92 ± 0.11	0.95 ± 0.07	0.61 ± 0.04	1.30 ± 0.08	0.71 ± 0.05	1.32 ± 0.19
Phosphorus	0.04 ± 0.00	0.10 ± 0.01	0.18 ± 0.02	0.31 ± 0.06	0.60 ± 0.08	0.85 ± 0.03
Potassium	0.75 ± 0.03	0.56 ± 0.02	0.54 ± 0.02	2.43 ± 0.08	0.34 ± 0.01	1.58 ± 0.04
Sodium	3.35 ± 0.08	4.08 ± 0.15	2.32 ± 0.05	0.04 ± 0.01	2.07 ± 0.12	3.64 ± 0.03
Trace elements (µg/g)						
Cobalt	ND	4.4 ± 0.1	6.7 ± 0.2	ND	7.9 ± 0.1	2.9 ± 0.0
Copper	77.4 ± 3.8	27.1 ± 1.2	32.5 ± 1.5	96.1 ± 3.6	60.1 ± 4.4	35.7 ± 2.1
Iron	288.8 ± 4.6	393.3 ± 2.8	713.9 ± 12.1	1215.7 ± 28.6	454.8 ± 8.2	1051.3 ± 12.8
Manganese	11.2 ± 0.2	42.7 ± 1.4	49.7 ± 0.4	38.6 ± 0.6	17.5 ± 0.8	122.2 ± 3.5
Zinc	356.4 ± 6.7	852.1 ± 15.5	1169.0 ± 18.9	622.3 ± 4.8	1081.3 ± 12.9	23.9 ± 0.6
Toxins (µg/g)^a^						
Cadmium	0.09 ± 0.01	0.06 ± 0.01	0.07 ± 0.00	ND	0.19 ± 0.02	ND
Na/K	4.51 ± 0.11	7.18 ± 0.23	4.29 ± 0.17	0.02 ± 0.00	5.99 ± 0.08	2.43 ± 0.06
Ca/P	28.50 ± 0.25	27.82 ± 1.78	25.33 ± 1.71	7.89 ± 0.88	5.46 ± 0.24	1.2 ± 0.06

*ND* not detected

^a^Toxic elements: selenium, vanadium, chromium and lead were lower than detection. All data are averages of biological triplicates ± standard deviation

The maximum contents of individual minerals and trace elements occurred at different MSGR concentrations. The calcium content ranged from 1.14% (1/1000 MSGR) to 4.58% (1/200 MSGR), which was higher than the content of 1% for mZM. Besides the 2.92% level of magnesium in the 1/1000 run, *S. subsalsa* in other seawater media accumulated less Mg than that in mZM. Regarding trace elements, the highest levels of Cu and Fe occurred in 1/100 MSGR medium, Co in 1/50 medium, and Zn in 1/200 medium.

The Ca:P ratio is an important index of nutritional value. The principle component of animal bone is hydroxyapatite (Ca_5_(PO_4_)_3_(OH)), where the Ca:P ratio is 2.15:1. Nutritional experts recommended 2:1 as the most suitable dietary Ca:P ratio for young rapidly growing farmed livestock and fish, even optimally 1:1 [21]. However, much higher ratios (e.g., 12:1) are demanded by mature production animals (e.g., laying chickens, dairy cattle) for calcium-rich material development [22]. Modified Zarrouk medium produced *Spirulina* biomass with well-balanced Ca:P ratios (1.2 ± 0.06), which are consistent with reports for commercially established *Spirulina* species (1.0–1.7:1) summarized by Tibbetts et al. [16] and perfect for young animals. Mature production animals might like the algal biomass from seawater media, where the lowest Ca:P ratio ranged from 7.89:1 (1/100 MSGR) to 28.50:1 (1/1000 MSGR). The high Ca:P ratios resulted from low supply of phosphorus by seawater and MSGR.

As feed or fertilizer, toxic elements must be a priori consideration for nutritional and safety evaluations. Fortunately, as given in Table 2, all measured heavy metal concentrations (e.g., As, Cd, Pb) in *S. subsalsa* produced in seawater and MSGR met the standard limits for safe consumption as feeds (As ≤ 0.5 µg/g; Cd ≤ 0.2 µg/g; Pb ≤ 2 µg/g) [18].

### *Spirulina subsalsa* in baggy reactor with reused medium

The above trials show that seawater plus MSGR is an efficient cultivation system to produce biofuel feedstock in laboratory. However, more analysis and trials are needed to confirm if this method can be economically viable large scale to realize the goal of applying algal technology to combat the global energy crisis.

To construct an offshore cyanobacteria site, infrastructure such as water piping, reactor and material transportation is an inevitable and undifferentiated investment independent of medium. However, in terms of water and nutrient input, seawater-MSGR would cost less compared to mZM. Seawater costs significantly less than the fresh water used in mZM due to its huge reserves and simple pretreatment. In terms of nutrients, it costs approximately 1 CNY to prepare one liter of mZM media. Considering total nutrients of 22.138 g/L in mZM and 5 mL/L in 1/200 MSGR, using MSGR is as feasible as using the compounds needed for mZM media. The generation of MSGR, the nutrients in seawater medium, did not cause any negative effect on economy. Seawater added with MSGR could be granted as a low-cost medium and possibly environmentally friendly system for producing cyanobacteria biomass.

To consider the potential of scaleup, we used a bag bioreactor to limit evaporation and contamination and ensure the sustainable operation of this system, as we discussed in our previous report [8]. Based on the best growth and lipid yield in 1/200 MSGR and maximizing the value of wastewater, the media used in this trial were prepared with seawater added by MSGR in a ratio of 1/200 (*V*_MSGR_/*V*_seawater_).

**Fig. 3:**
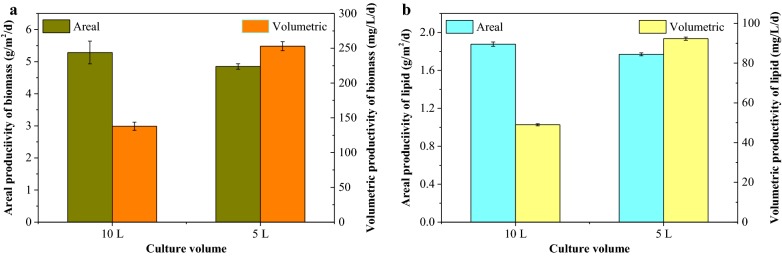
Biomass (**a**) and lipid (**b**) productivity of *Spirulina subsalsa* grown in different volume of seawater supplemented with 1/200 MSGR (*V*_MSGR_/*V*_seawater_). All data are averages of biological triplicates ± standard deviation

In large-scale production, areal and/or volumetric productivity are the main parameters to consider for space and cost efficiency [23, 24]. These biomass and lipid productivities of different medium volumes are shown in Fig. 3. The 10-L system produced higher biomass and lipid per unit of area than the 5-L one, while the 5-L work volume exhibited superiority when considering the biomass and lipid productivity per unit of medium. The 5-L volume had almost twice higher biomass (1.3 g/L) than 0.7 g/L in 10-L system. The height in 10-L reactor led to that result, because the reduced light to cyanobacterial cells at the bottom limited their growth.

**Fig. 4:**
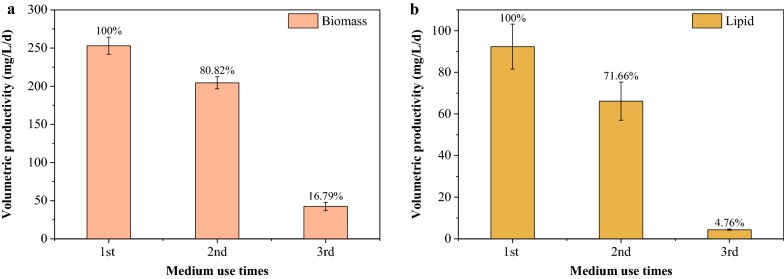
Biomass (**a**) and lipid (**b**) productivity of *Spirulina subsalsa* grown in reused seawater supplemented with 1/200 MSGR (*V*_MSGR_/*V*_seawater_). Percentages on the bar represent the ratio compared with the value obtained from the first use medium. All data are averages of biological triplicates ± standard deviation

To maximize biomass production per unit of seawater and minimize the input, we reused the spent seawater medium. After culturing *S. subsalsa*, limited phosphorus (< 0.05 mg/L) remained in spent medium, so 1/200 MSGR was supplemented to supply nutrients before reuse. The growth and lipid accumulation of *S. subsalsa* in different times of reused medium are shown in Fig. 4. Both measurements decrease with each additional reuse. It was hard for *S. subsalsa* to survive in the third-use medium, but the second use of seawater obtained around 81% of biomass yield (Fig. 4a) and 72% of lipid yield (Fig. 4b), compared to the productivity from the first use of medium. Reusing medium once obtained 2.3 g biomass from one liter of seawater and twice got 2.5 g/L, while the former had a biomass productivity of 229 mg/L/day and the latter decreased to 167 mg/L/day. Reusing medium can efficiently raise the output of biofuel feedstock from a certain volume of seawater; however, the productivity decreased with every reuse. Our results suggest that one reuse of seawater is the best condition to obtain a high biomass production per liter of seawater with a high productivity simultaneously.

Moreover, the cultivation with reused medium did not significantly change the fatty acid profiles, and the six properties suggest that all the biomass could produce biodiesel matching the ASTMD and EN standard (Table 3).

**Table 3:** The fatty acid profile of *Spirulina subsalsa* in reused seawater supplemented with 1/200 MSGR (*V*_MSGR_/*V*_seawater_)

	First	Second	ASTMD 6751-08	EN 14214
Fatty acids (% total FAME)				
C12:0 (lauric)	11.48 ± 0.62	2.77 ± 0.26		
C14:0 (myristic)	1.25 ± 0.03	3.55 ± 0.43		
C16:0 (palmitic)	35.50 ± 0.59	33.96 ± 0.77		
C16:1 (palmitoleic)	17.83 ± 0.78	23.76 ± 0.81		
C18:0 (stearic)	2.76 ± 0.31	4.94 ± 0.61		
C18:1 (oleic)	14.51 ± 0.50	15.78 ± 0.86		
C18:2 (linoleic)	14.28 ± 0.56	12.30 ± 0.97		
Others	2.40 ± 1.76	2.93 ± 1.87		
Biodiesel properties				
Kinematic viscosity 40 °C (mm^2^/s)	4.82 ± 0.01	4.80 ± 0.01	1.9–6.0	3.5–5.0
Specific gravity (kg/L)	0.88 ± 0.00	0.88 ± 0.00	0.85–0.90	0.86–0.90
CP (°C)	11.86 ± 0.11	11.42 ± 0.27		
CN	58.82 ± 0.06	58.60 ± 0.13	≥ 47	≥ 51
IV (gI_2_/100 g)	57.99 ± 0.62	60.41 ± 1.48		51–120
HHV (MJ/kg)	39.61 ± 0.01	39.66 ± 0.03		

All data are averages of biological triplicates ± standard deviation

**Fig. 5:**
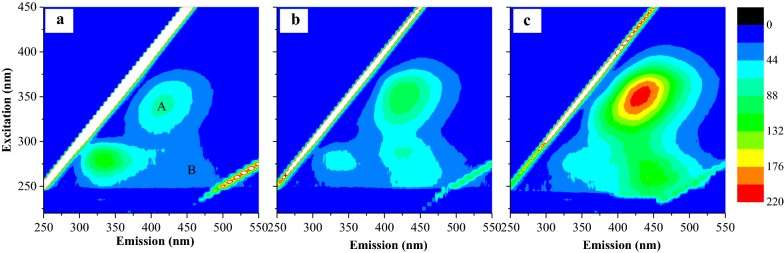
Excitation-emission matrix fluorescence spectra of *Spirulina subsalsa* medium containing seawater and MSGR, at the beginning of **a** first use, **b** second use and **c** third use. Region A: humic-like substances, Region B: fulvic-like substances

To understand the limitations on reusing medium, the excitation–emission matrix fluorescence spectra of medium were observed at the beginning of every culture period (Fig. 5). Comparing with the medium in first two uses, the intensity indicating humic- and fulvic-like substances (reported toxic to algae [25]) increased in the third use of the media. This might be the main problem for cyanobacterial survival when reusing medium several times. Continuously reusing seawater could be feasible via partial supplement with fresh seawater to decrease the inhibitor concentration or degrading the inhibitor though catalysis, sonication, filtration or other physical/chemical methods.

Based on the above results, we propose some preferences about designing cultivation system of *S. subsalsa* in different places. For locations with limited space but ample seawater, the cultivation system can be built with a high height and without designing seawater reuse for producing areal biomass fast and efficiently, because fresh seawater is easy to access but medium reuse decreases biomass productivity. This cultivation style of low culture height might be employed by locations with enough space but high expenditure of getting seawater where the limitation is the seawater supply and obtaining as much biomass from per volume of seawater as possible can improve the economic feasibility of the production process. Moreover, seawater medium reuse was shown as an applicable way to achieve this goal.

### Response and adaption of *Spirulina subsalsa* to seawater

As a halotolerant cyanobacterium, *S. subsalsa* is easily adapted to hypersaline and alkaline environments [26]. The growth rate and chlorophyll content of the cells were enhanced in seawater and MSGR media (Fig. 1), suggesting that the organism has a strong adaptation to deal with the salinity in seawater. The main effect of high salinity on *S. subsalsa* cells is osmotic stress that decreases cytosol moisture and increases intracellular salt level. Major aspects addressed by salt stress include the regulation of membrane, osmotic accumulation through organic osmolytes and inorganic ions, and sodium exclusion [27].

#### Regulation of membrane

**Fig. 6:**
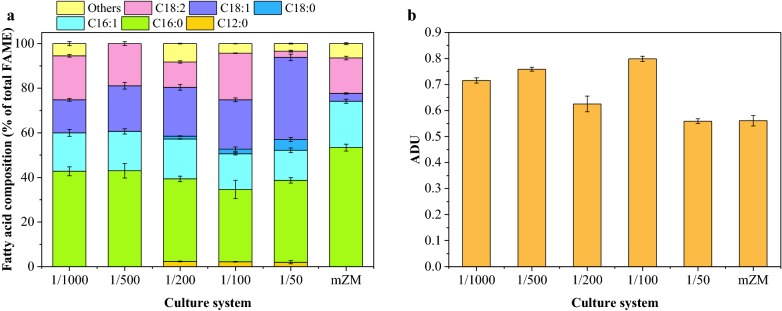
Fatty acid profiles (**a**) and fatty acid indices (**b**) for *Spirulina subsalsa* grown in mZM, in seawater supplemented with MSGR in different volume ratios (1/1000, 1/500, 1/200, 1/100 and 1/50, *V*_MSGR_/*V*_seawater_). *FAME* fatty acid methyl ester, *ADU* average degree of unsaturation of fatty acids. All data are averages of biological triplicates ± standard deviation

The fluidity of the cell membrane is changed through its fatty acid tails to deal with abiotic stress [28]. Fatty acid (FA) profiles of *S. subsalsa* cultivated in mZM, seawater with different loadings of MSGR are shown in Fig. 6a. The characteristics of the major fatty acids recorded in this study were in accordance with previous reports [29], including high percentage in C16:1 and lack of C18:3. The fatty acid composition in cyanobacterial biomass from freshwater media (mZM) is predominantly palmitic acid (C16:0). In general, seawater treatment resulted in a remarkable decrease of saturated palmitic acid and increase of unsaturated fatty acids, especially monounsaturated palmitoleic acid (MUFA) (Fig. 6a, b). This may result from enhanced desaturation and elongation reactions of the saturated fatty acids in *S. subsalsa* under seawater and MSGR cultivation. Increased elongation and desaturation of fatty acid are causally linked to salt tolerance of microalgae and cyanobacteria through protecting the integrity of membrane and its many ion channels and transport systems [28, 30]. Although data in Fig. 6 were from total fatty acids in *S. subsalsa* cells, they may also indicate the change of fatty acids located on the membrane. To achieve a rigid cell structure under such a new environment, *S. subsalsa* reduced its membrane fluidity and permeability via accumulation of oleic acid (C18:1) in its membrane, compared with that in mZM. Moreover, the MUFA proportion increased from 32 to 50% with an increase in the ratio of MSGR to seawater from 1/1000 to 1/50, which indicated that the supplement of this wastewater could help *S. subsalsa* deal with salinity in seawater.

#### Osmotic response

**Fig. 7:**
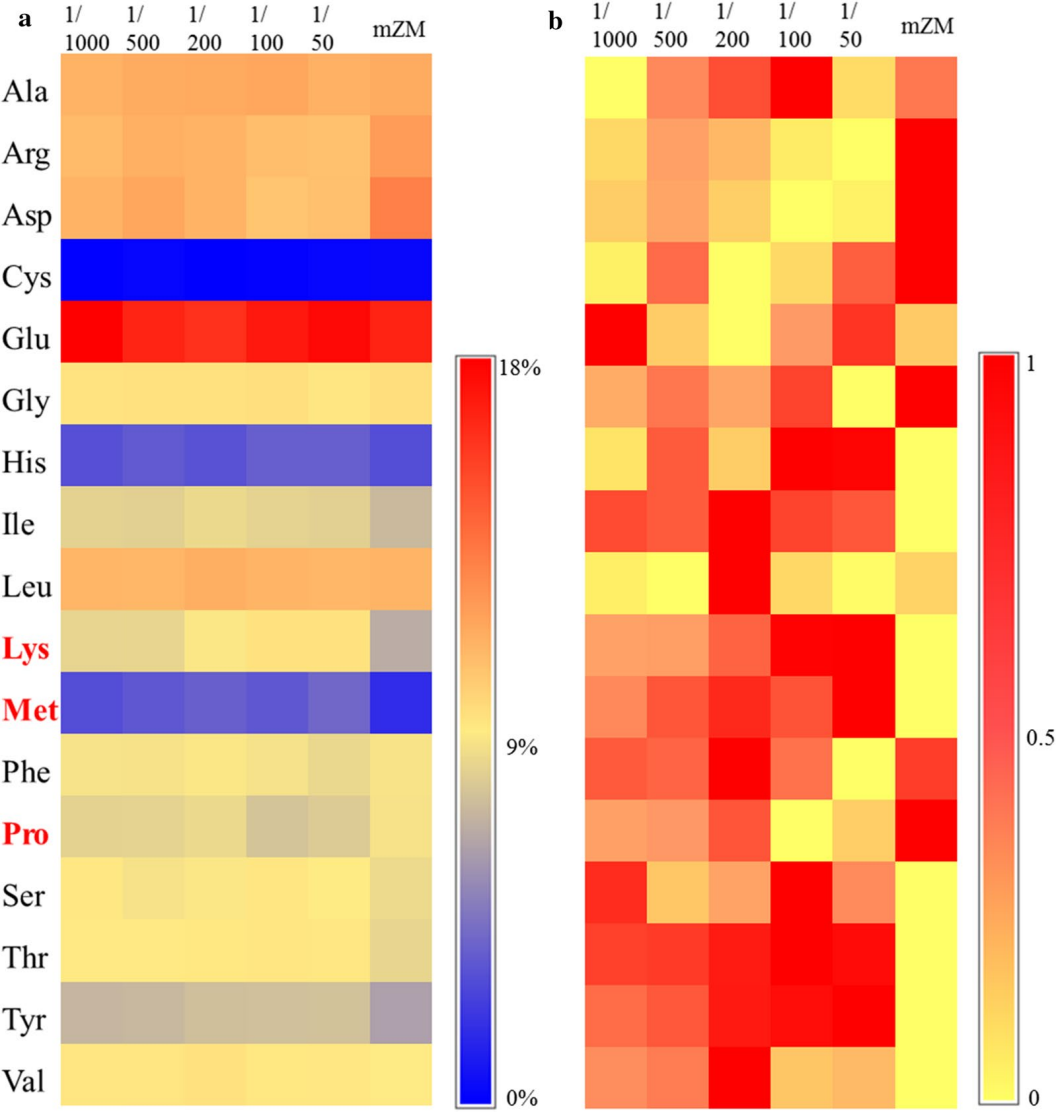
Amino acid profiles for *Spirulina subsalsa* grown in mZM, in seawater supplemented with MSGR in different volume ratios (1/1000, 1/500, 1/200, 1/100 and 1/50, *V*_MSGR_/*V*_seawater_). The unit in **a** is the percentage of total amino acids. The data in **b** is normalized by inter control from **a**

In response to environmental stress, plants and algae commonly tend to accumulate some amino acids as osmolytes, including proline, lysine, and their downstream metabolites (lysine-mediated betaine) [31–33]. The content of 17 kinds of amino acids is shown in Fig. 7. *Spirulina subsalsa* accumulates more glutamic acid than any other amino acid, making up approximately 15% of total amino acids (TAA). Cysteine accumulated in the lowest concentration, ranging from 0.3 to 0.4% of TAA (Fig. 7a). Because there is a huge difference in concentration among different amino acids, we normalized the amino acids contents by internal control to compare the change of each amino acid in different media (Fig. 7b).

**Fig. 8:**
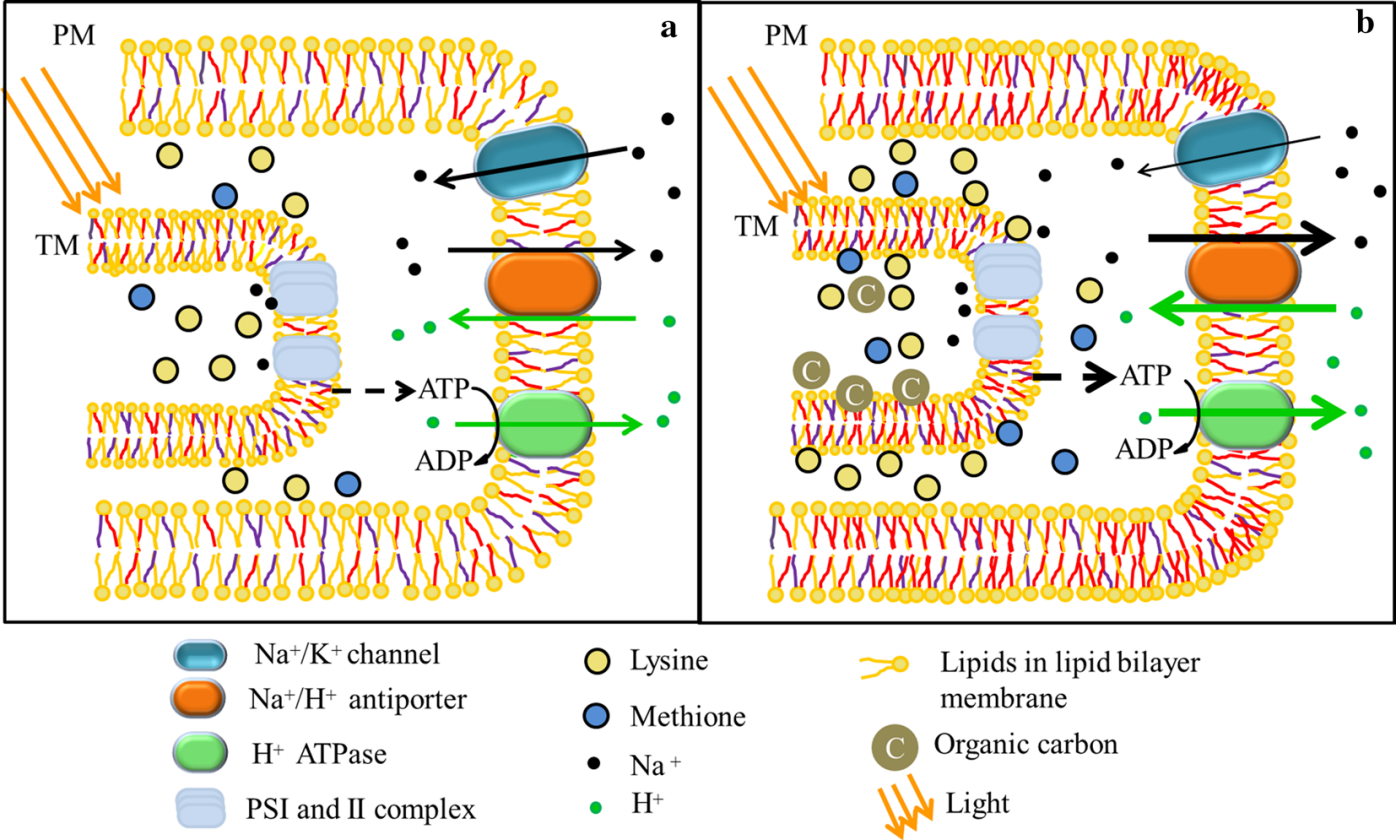
A simplified scheme showing regulation employed by *Spirulina subsalsa* to survive in mZM (**a**) and seawater mixed with MSGR (**b**). Different color of tails for lipids in membrane stands for the saturability of fatty acids: yellow is for saturated tail, red colored monosaturated tail, purple represents polysaturated tail. The width of the arrows represents the speed of ions moving towards the arrow direction. PM: plasma membrane; TM: Thylakoid membrane; ATP: adenosine triphosphate; ADP: adenosine diphosphate

Among amino acids and proteins that are induced by salt or drought stress, proline has been studied most extensively [33]. Compared with the sample from freshwater-prepared media (mZM), the cyanobacteria from seawater media produced around 1.3 times more lysine and 1.8 times more methionine, and less proline (highlighted in Fig. 7 and visualized in Fig. 8). Unlike the species that depend on proline to deal with the salt stress, *Spirulina subsalsa* involved increased lysine and methionine content responding to salinity in seawater media.

Lysine is an important metabolite that has stress-associated functions. It protects cells from salt or desiccation stress both as an osmolyte and via enzymatic protection. Betaine substances, derived from lysine, acts as an efficient osmolyte in plant cells to adjust the osmotic pressure and stabilize other macromolecular materials [34]. The lysine-rich protein in *Craterostigma plantagineum* promoted phosphatidic acid binding and protected enzymes from adverse effects of desiccation [35]. Wang et al. [36] also manifested that the maize mutant with increased lysine and protein content had a significantly stronger salt resistance. The increased lysine content indicated that *Spirulina subsalsa* might also protect itself from salinity by accumulating lysine-related protein or other organic substances.

Methionine (Met) is also involved in abiotic response as a compatible osmolyte [37]. In this study, the cyanobacterium in seawater media (1.01–1.15% dry mass in 1/200, 1/100 and 1/50 MSGR) accumulated twice as much Met and Met-derived substances than that in mZM (0.52–0.56% of dry mass). This might result from high salinity and vitamin B12 in seawater. The activity of Met synthase can be promoted by the addition of vitamin B12 [38], and seawater was reported to contain a certain of vitamin B12 [39]. Salt stress-induced Met synthesis and vitamin ensured the reaction activity.

#### Sodium exclusion

In a saline environment, cells usually experience an intracellular sodium upshock due to passive diffusion. To survive in this upshock, the *Spirulina* cells must possess some suitable mechanisms to exclude sodium. All runs in seawater media (except in the 1/500 media) had the same or lower amount of sodium content in the cells as the mZM control (Table 2), indicating that *S. subsalsa* possesses a strong capability of sodium exclusion to deal with high salinity.

Organisms secrete sodium out through Na^+^/K^+^ channels and Na^+^/H^+^ antiport system, consisting of Na^+^/H^+^ antiporter(s) and H^+^ ATPase(s) [30]. These two systems are located in the plasma membrane and are influenced by the profile of fatty acids, as shown in Fig. 8.

The first adjustment was usually compensated by potassium uptake and depressed by the unsaturation of fatty acids [40, 41], shown as the thinning arrow on the Na^+^/K^+^ channel in Fig. 8b. Therefore, halotolerant organisms usually have low Na^+^/K^+^ ratios in saline media. However, aside from 1/100 MSGR medium, cyanobacterial cells in most seawater treatments exhibited the Na^+^/K^+^ ratios ranging from 4.3 to 7.3, which were higher than the ratio of 2.3 for mZM. *S. subsalsa* may not depend a lot on this process coupled with enhanced K uptake due to the increased average degree of unsaturation (ADU) in seawater media (Fig. 6b).

With respect to unsaturated fatty acids, the second regulation method worked harmoniously with membrane protection, because the increased ADU can trigger the synthesis of the Na^+^/H^+^ antiporter and H^+^ ATPase, shown as the thicker arrows on Na^+^/H^+^ antiporter(s) and H^+^ ATPase in Fig. 8b, compared with that in Fig. 8a. The lower pH values in seawater media than in mZM, as shown in Additional file 1: Fig. S5, also proved that the H^+^ ATPase might work well. Indeed, this process can only be created and maintained at the expense of energy that would be provided by photosynthesis and respiration. The production and distribution of energy in cyanobacterial cells likely influenced the intracellular ion activity in seawater media at various MSGR concentrations. For *S. subsalsa* cultivated in seawater and MSGR media, the energy from photosynthesis could be reflected by the chlorophyll a content and that from respiration was tied up in the organic carbon mainly provided by MSGR.

The aforementioned obvious sodium exclusion by *S. subsalsa* suggested that the energy flowing into the Na-excluding process was sufficient, although chl a content decreased in 1/1000, 1/500 and 1/50 MSGR media (Fig. 1). The similar chl a content between 1/200 and 1/100 treatments was accompanied by a huge difference in Na/K ratio (4.30 and 0.02, respectively). These results indicate the contribution of respiratory energy to this ion exchange process. The respiratory activity is markedly enhanced in the presence of an exogenous electron donor, such as the phosphorylation substrates ADP, phosphate, and magnesium [42]. For heterotrophic *S. subsalsa*, organic material can also be an exogenous electron donor benefiting respiratory energy production. Compared to the 1/200 group, the higher carbon concentration occurred with the 1/100 and 1/50 groups (Additional file 1: Fig. S6), providing more energy for ion activity, leading to less Na^+^ in cell (Fig. 8). In this cultivation system consisting of seawater and wastewater, *S. subsalsa* excluded sodium mainly through Na^+^/H^+^ antiport system that depended on fatty acids and energy produced by photosynthesis and organic carbon respiration.

In all, the three methods relating to membrane regulation, osmolytes adjustment, and sodium exclusion were all employed by *S. subsalsa* to survive and grow well in seawater. The adjustment of fatty acids maintained the fluidity and integrity of the membrane and its signal channels and transporters. The accumulation of amino acids balanced osmotic stress and protected the cellular enzyme system. The secretion of sodium prohibited the unlimited increase of intracellular sodium. Indeed, to make the whole system work well, the help from the nutrients in MSGR was indispensable.

## Conclusion

This work indicates the feasibility of *S. subsalsa* cultivation in seawater to which a feedstock of nutrient compounds (MSGR) has been added. MSGR added in seawater in ratios (1/1000–1/50) obtained equal or higher levels of biomass concentration compared to freshwater medium mZM, and the ratios of 1/200 and 1/100 performed best. Besides biomass production, *S. subsalsa* obtained two-fold higher lipid content (35%) in 1/200 MSGR than that in mZM. MSGR-supplemented seawater in baggy reactors also produced *S. subsalsa* with a high biomass productivity (253 mg/L/day) and lipid generation (92 mg/L/day). Moreover, this medium can be reused once to achieve another 81% of biomass yield and 72% of lipid yield. *S. subsalsa* exhibits a powerful ability to tolerate high salinity in seawater through elongation and desaturation of fatty acids, accumulation of lysine and methionine, and secretion of sodium. The exclusion of sodium might be associated with Na^+^/H^+^ antiporter and H^+^ ATPase that depend on the energy from photosynthesis and respiration of organic carbon. This cultivation method using both seawater and MSGR could be a cost-effective way to produce *S. subsalsa* as the source of biofuel feedstock and has the potential to be amplified with large-scale baggy reactors in practice.

## Additional file

**Additional file 1: Fig. S1.** Schematic figure of *Spirulina subsalsa* production with seawater and MSGR: (a) flask bench to optimize the addition of MSGR for cell growth; (b) baggy reactor trial with reused seawater as a preliminary step of scaling up; (c) photo of baggy reactor. **Fig. S2.** Carbohydrate accumulation for *Spirulina subsalsa* grown in mZM and in seawater supplemented with MSGR in different volume ratios (1/1000, 1/500, 1/200, 1/100 and 1/50, *V*_MSGR_/*V*_seawater_). All data are averages of biological triplicates ± standard deviation. **Fig. S3.** Microscopy images of *Spirulina subsalsa* grown in seawater supplemented with MSGR in different volume ratio (*V*_MSGR_/*V*_seawater_; (a) 1/1000, (b) 1/500, (c) 1/200, (d) 1/100, (e) 1/50, (f) 1/25, (g) 1/10), in (f) mZM. The blue round cycled bacterium. Scale bar, 20 µm. **Fig. S4.** Microscopy images of *Spirulina subsalsa* grown in seawater supplemented with 1/50 MSGR (*V*_MSGR_/*V*_seawater_). Scale bar, 20 µm. **Fig. S5.** The pH value of *Spirulina subsalsa* culture from mZM, and from seawater supplemented with MSGR in different volume ratios (1/1000, 1/500, 1/200, 1/100 and 1/50, *V*_MSGR_/*V*_seawater_). All data are averages of biological triplicates ± standard deviation. **Fig. S6.** Total organic carbon in mZM, in seawater supplemented with MSGR in different volume ratios (1/1000, 1/500, 1/200, 1/100 and 1/50, *V*_MSGR_/*V*_seawater_) for cultivating *Spirulina subsalsa*. All data are averages of biological triplicates ± standard deviation.

## References

[1] Mata TM, Martins AA, Caetano NS (2010). Microalgae for biodiesel production and other applications: a review. Renew Sustain Energy Rev.

[2] Georgianna DR, Mayfield SP (2012). Exploiting diversity and synthetic biology for the production of algal biofuels. Nature.

[3] Wu Y, Hu H, Yu Y, Zhang T, Zhu S, Zhuang L, Zhang X, Lu Y (2014). Microalgal species for sustainable biomass/lipid production using wastewater as resource: a review. Renew Sustain Energy Rev.

[4] Markou G, Georgakakis D (2011). Cultivation of filamentous cyanobacteria (blue-green algae) in agro-industrial wastes and wastewaters: a review. Appl Energy.

[5] Rawat I, Kumar RR, Mutanda T, Bux F (2013). Biodiesel from microalgae: a critical evaluation from laboratory to large scale production. Appl Energy.

[6] Ho SH (2015). Dynamic metabolic profiling of the marine microalga *Chlamydomonas* sp. JSC4 and enhancing its oil production by optimizing light intensity. Biotechnol Biofuels.

[7] Chinnasamy S, Bhatnagar A, Hunt RW, Das KC (2010). Microalgae cultivation in a wastewater dominated by carpet mill effluents for biofuel applications. Bioresour Technol.

[8] Pei H, Jiang L (2018). Mixing seawater with a little wastewater to produce bioenergy from limnetic algae. Trends Biotechnol.

[9] Lin H (1991). Comparison of* Spirulina subsalsa* with other* Spirulina* species. Acta Hydrobiologica Sinica..

[10] Jiang L (2015). The feasibility of using complex wastewater from a monosodium glutamate factory to cultivate *Spirulina subsalsa* and accumulate biochemical composition. Bioresour Technol..

[11] Pei H, Jiang L, Hou Q, Yu Z (2017). Toward facilitating microalgae cope with effluent from anaerobic digestion of kitchen waste: the art of agricultural phytohormones. Biotechnol Biofuels.

[12] Song M, Pei H, Hu W, Ma G (2013). Evaluation of the potential of 10 microalgal strains for biodiesel production. Bioresour Technol..

[13] GB50095. Determination of protein in foods. National food safety standard. 2010.

[14] Lourenço SO, Barbarino E, Lavín PL, Marquez UML, Aidar E (2004). Distribution of intracellular nitrogen in marine microalgae: calculation of new nitrogen-to-protein conversion factors. Eur J Phycol.

[15] Shao Y, Pan J, Zhang C, Jiang L, He Y (2015). Detection in situ of carotenoid in microalgae by transmission spectroscopy. Comput Electron Agric.

[16] Tibbetts SM (2015). Biochemical characterization of microalgal biomass from freshwater species isolated in Alberta, Canada for animal feed applications. Algal Res.

[17] Matos ÂP (2016). Chemical characterization of six microalgae with potential utility for food application. J Am Oil Chem Soc.

[18] GB16919. Food grade *Spirulina* powder. National food safety standard. 1997.

[19] Radulović N, Stojanović G, Palić R (2006). Composition and antimicrobial activity of *Equisetum arvense* L. essential oil. Phytother Res..

[20] Singh R (2017). Uncovering potential applications of cyanobacteria and algal metabolites in biology, agriculture and medicine: current status and future prospects. Front Microbiol..

[21] National Research Council (2011). Nutrient requirements of fish and shrimp.

[22] McDonald P, Edwards RA, Greenhalgh JFD, Morgan CA, Sinclair LA (2010). Animal nutrition.

[23] Singh RN, Sharma S (2012). Development of suitable photobioreactor for algae production—a review. Renew Sustain Energy Rev.

[24] Li Q, Powers W, Rozeboom D, Liu Y, Liao W (2016). An integrated Water Curtain-Microalgal Culture system (WCMC) to mitigate air emissions and recover nutrients from animal feeding operations. Algal Res.

[25] Lin D, Ji J, Long Z, Yang K, Wu F (2012). The influence of dissolved and surface-bound humic acid on the toxicity of TiO_2_ nanoparticles to *Chlorella* sp. Water Res.

[26] Gabbay-Azaria R, Tel-Or E (1991). Regulation of intracellular Na^+^ content during NaCl upshock in the marine cyanobacterium *Spirulina subsalsa* cells. Bioresour Technol..

[27] Farmer EE, Almeras E, Krishnamurthy V (2003). Jasmonates and related oxylipins in plant responses to pathogenesis and herbivory. Curr Opin Plant Biol.

[28] Azachi M, Sadka A, Fisher M, Goldshlag P, Gokhman I, Zamir A (2002). Salt induction of fatty acid elongase and membrane lipid modifications in the extreme halotolerant alga *Dunaliella salina*. Plant Physiol.

[29] Cohen Z, Vonshak A, Richmond A (1987). Fatty acid composition of *Spirulia* strains grown under various environmental conditions. Phytochemistry.

[30] Singh SC, Sinha RP, Hader DP (2002). Role of lipids and fatty acids in stress tolerance in cyanobacteria. Acta Protozool..

[31] Galili G, Tang G, Zhu X, Gakiere B (2001). Lysine catabolism: a stress and development super-regulated metabolic pathway. Curr Opin Plant Biol.

[32] Sharma SS, Dietz KJ (2006). The significance of amino acids and amino acid-derived molecules in plant responses and adaptation to heavy metal stress. J Exp Bot.

[33] Szabados L, Savoure A (2009). Proline: a multifunctional amino acid. Trends Plant Sci.

[34] Ashrafa M, Foolad MR (2007). Roles of glycine betaine and proline in improving plant abiotic stress resistance. Environ Exp Bot.

[35] Petersen J, Eriksson SK, Harryson P, Pierog S, Colby T, Bartels D, Röhrig H (2012). The lysine-rich motif of intrinsically disordered stress protein CDeT11-24 from *Craterostigma plantagineum* is responsible for phosphatidic acid binding and protection of enzymes from damaging effects caused by desiccation. J Exp Bot.

[36] Wang M, Liu C, Li S, Zhu D, Zhao Q, Yu J (2013). Improved nutritive quality and salt resistance in transgenic maize by simultaneously overexpression of a natural lysine-rich protein gene, *SBgLR*, and an ERF transcription factor gene, *TSRF1*. Int J Mol Sci.

[37] Joshi V, Joung J, Fei Z, Jander G (2010). Interdependence of threonine, methionine and isoleucine metabolism in plants: accumulation and transcriptional regulation under abiotic stress. Amino Acids.

[38] Croft MT, Lawrence AD, Raux-Deery E, Warren MJ, Smith AG (2005). Algae acquire vitamin B12 through a symbiotic relationship with bacteria. Nature.

[39] Okbamichael M, Sañudo-Wilhelmy SA (2004). A new method for the determination of vitamin B12 in seawater. Anal Chim Acta.

[40] Oren A (2003). Intracellular salt concentrations and ion metabolism in halophilic microorganisms. Halophilic Microorg Environ.

[41] Allakhverdiev SI, Kinoshita M, Inaba M, Suzuki I, Murata N (2001). Unsaturated fatty acids in membrane lipids protect the photosynthetic machinery against salt induced damage in *Synechococcus*. Plant Physiol.

[42] Gabbay-Azaria R, Tel-Or E (1985). Cyanobacterial biomass production in saline media. Plant Soil.

