# Supplementary material for: Filamentous cyanobacteria triples oil production in seawater-based medium supplemented with industrial waste: monosodium glutamate residue
Source: Biotechnol Biofuels. 2019 Mar 14;12:53. doi: 10.1186/s13068-019-1391-1 (PMC6417114; doi:10.1186/s13068-019-1391-1)
Supplement: Supplementary file 1 — Additional file 1: Fig. S1. Schematic figure of Spirulina subsalsa production with seawater and MSGR: (a) flask bench to optimize the addition of MSGR for cell growth; (b) baggy reactor trial with reused seawater as a preliminary step of scaling up; (c) photo of baggy reactor. Fig. S2. Carbohydrate accumulation for Spirulina subsalsa grown in mZM and in seawater supplemented with MSGR in different volume ratios (1/1000, 1/500, 1/200, 1/100 and 1/50, VMSGR/Vseawater). All data are averages of biological triplicates ± standard deviation. Fig. S3. Microscopy images of Spirulina subsalsa grown in seawater supplemented with MSGR in different volume ratio (VMSGR/Vseawater; (a) 1/1000, (b) 1/500, (c) 1/200, (d) 1/100, (e) 1/50, (f) 1/25, (g) 1/10), in (f) mZM. The blue round cycled bacterium. Scale bar, 20 µm. Fig. S4. Microscopy images of Spirulina subsalsa grown in seawater supplemented with 1/50 MSGR (VMSGR/Vseawater). Scale bar, 20 µm. Fig. S5. The pH value of Spirulina subsalsa culture from mZM, and from seawater supplemented with MSGR in different volume ratios (1/1000, 1/500, 1/200, 1/100 and 1/50, VMSGR/Vseawater). All data are averages of biological triplicates ± standard deviation. Fig. S6. Total organic carbon in mZM, in seawater supplemented with MSGR in different volume ratios (1/1000, 1/500, 1/200, 1/100 and 1/50, VMSGR/Vseawater) for cultivating Spirulina subsalsa. All data are averages of biological triplicates ± standard deviation. [file 13068_2019_1391_MOESM1_ESM.docx]

**Additional information**

**List:**

**Fig. S1** Schematic figure of *Spirulina subsalsa* production with seawater and MSGR: (a) flask bench to optimize the addition of MSGR for cell growth; (b) baggy reactor trial with reused seawater as a preliminary step of scaling up; (c) photo of baggy reactor.

**Fig. S2** Carbohydrate accumulation for *Spirulina subsalsa* grown in mZM and in seawater supplemented with MSGR in different volume ratios (1/1000, 1/500, 1/200, 1/100 and 1/50, V_MSGR_/V_seawater_). All data are averages of biological triplicates ± standard deviation.

**Fig. S3** Microscopy images of *Spirulina subsalsa* grown in seawater supplemented with MSGR in different volume ratio (V_MSGR_/V_seawater_; a 1/1000, b 1/500, c 1/200, d 1/100, e 1/50, f 1/25, g 1/10), in (f) mZM. The blue round cycled bacterium. Scale bar, 20 µm.

**Fig. S4** Microscopy images of *Spirulina subsalsa* grown in seawater supplemented with 1/50 MSGR (V_MSGR_/V_seawater_). Scale bar, 20 µm.

**Fig. S5** The pH value of *Spirulina subsalsa* culture from mZM, and from seawater supplemented with MSGR in different volume ratios (1/1000, 1/500, 1/200, 1/100 and 1/50, V_MSGR_/V_seawater_). All data are averages of biological triplicates ± standard deviation.

**Fig. S6** Total organic carbon in mZM, in seawater supplemented with MSGR in different volume ratios (1/1000, 1/500, 1/200, 1/100 and 1/50, V_MSGR_/V_seawater_) for cultivating *Spirulina subsalsa*. All data are averages of biological triplicates ± standard deviation.


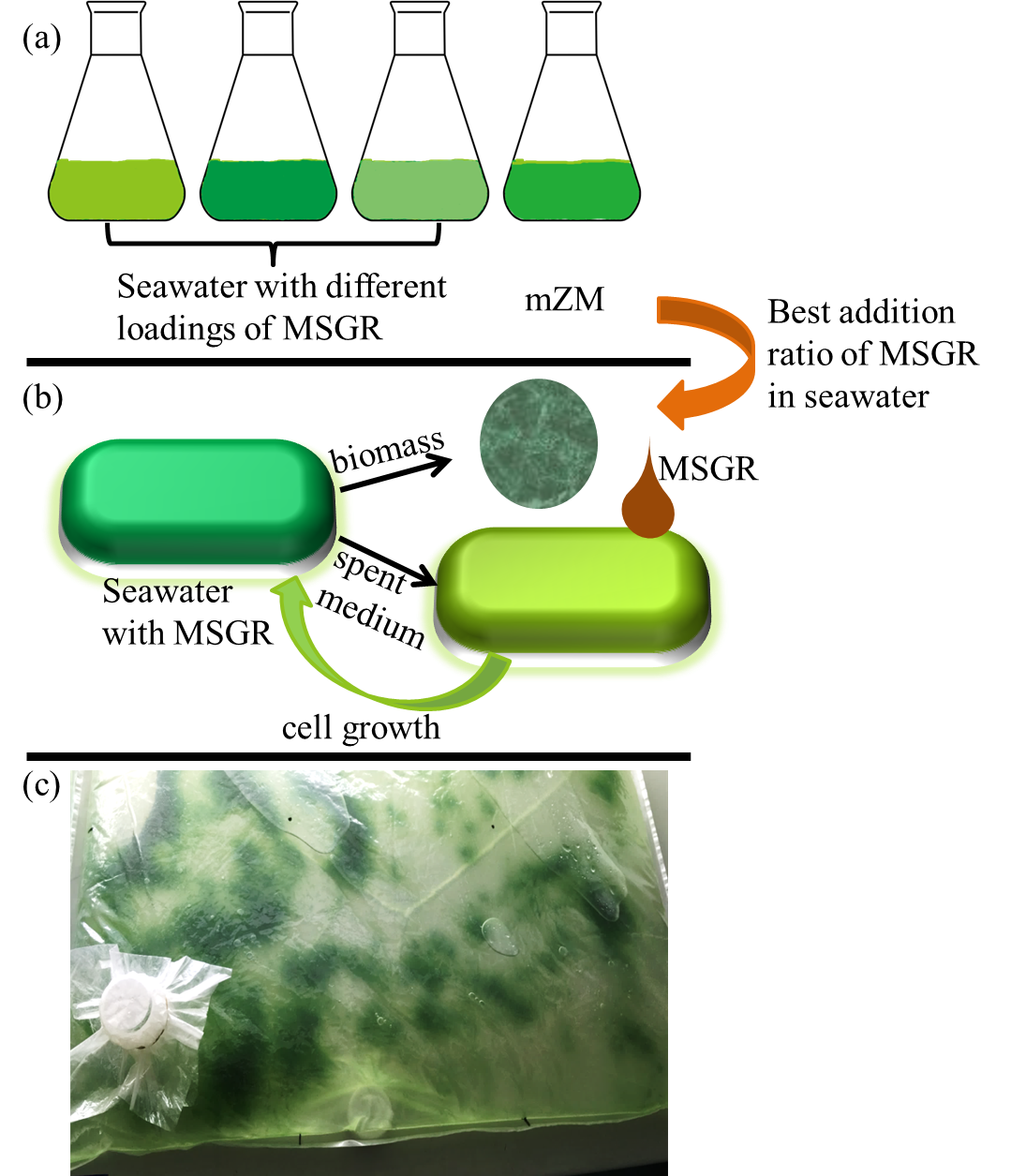


**Fig. S1**

**Fig. S2**

**Fig. S3**


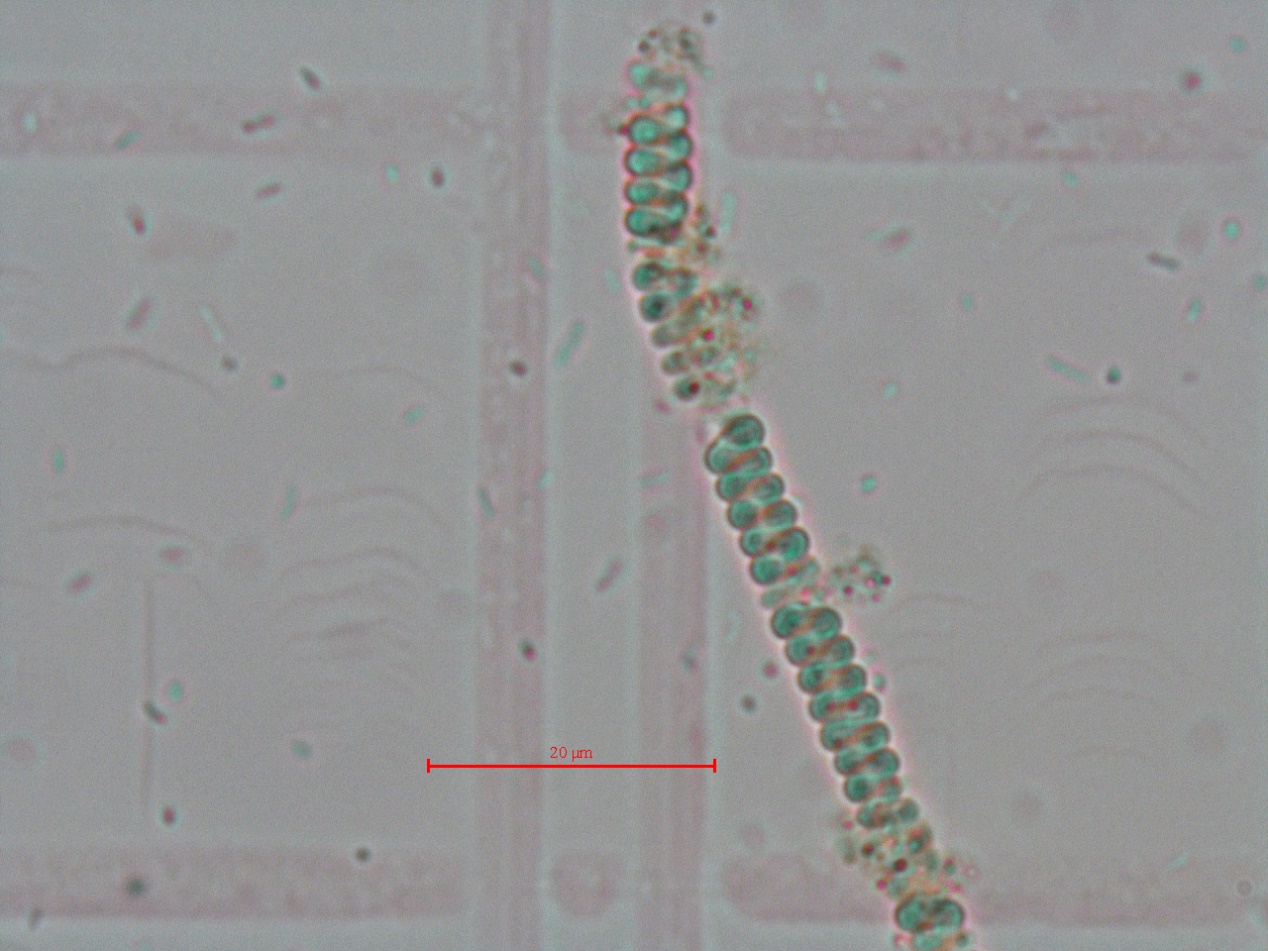


**Fig. S4**

**Fig. S5**

**Fig. S6**
